# Evaluation of the effect of *Nigella sativa* extract on human hepatocellular adenocarcinoma cell line (HepG2) *in vitro*

**DOI:** 10.1186/1471-2164-15-S2-P63

**Published:** 2014-04-02

**Authors:** Fazal Khan, Gauthaman Kalamegam, Mamdooh Gari, Adel Abuzenadah, Adeel Chaudhary, Mohammed Al Qahtani, Khalid Al Ghamdi, Tariq Jamal, Abdulrahman Al Malki, Taha Kumosani

**Affiliations:** 1 Department of Biochemistry, Faculty of Science, King Abdulaziz University, Kingdom of Saudi Arabia; 2 Stem Cell Unit, Centre of Excellence in Genomic Medicine Research, King Abdulaziz University, Kingdom of Saudi Arabia; 3 Department of Ear, Nose and Throat, Faculty of Medicine, King Abdulaziz University, Kingdom of Saudi Arabia; 4 Department of Medical Laboratory Technology, Faculty of Applied Medical Sciences, King Abdulaziz University, Kingdom of Saudi Arabia

## Background

**Figure 1:**
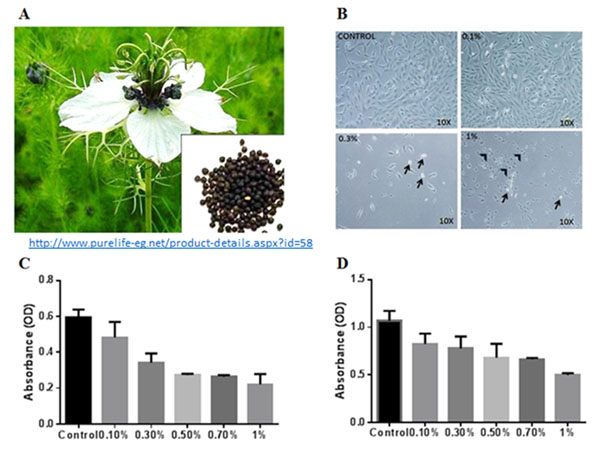
**(A).** Photograph of *Nigella sativa* plant, flower and seeds (inset). **(B)**. Phase contrast microscopic images showing the HepG2 cell morphology of control and treated groups (0.1%, 0.3% and 1% *Niegella sativa* extract at 24 h).The short black arrow indicates dead cells and the arrow head indicates cell shrinkage. **(C, D).** Cell proliferation (MTT assay) results of Hep G2 cell line following treatment with *Nigella sativa* extract at varying concentrations (0.1%, 0.3%,0.5%,0.7% and 1%) for 24 h (C) incubation and 48 h **(D)**. All values are expressed as mean ± SEM from three different replicates.

Cancer is a dreadful disease and remains a major cause of mortality world-wide. Plant derived compounds such as vincristine, vinblastine, etoposide, camptothecin etc. are widely used in cancer therapeutics. *Nigella sativa* (Figure 1) is claimed to have antihypertensive, analgesic, diuretic, anti-bacterial and liver protective effects [1]; however, there are only very few scientific evidence. In the present study, we attempt to explore the anticancer cancer claims of *Nigella sativa*, on human hepatocellular adenocarcinoma (HepG2) cell line *in vitro*.

## Materials and methods

The whole extract of *Nigella sativa* (generously donated by the ENT research group, KAUH) was filter sterilized using 0.2µm syringe filters. The HepG2 cells were seeded at 3 x 10^4^ cells/well of a 24-well tissue culture plate and cultured overnight in DMEM low glucose medium supplemented with 10% fetal bovine serum, 200mM GlutaMax, 1% penicillin/streptomycin under standard culture conditions of 37°C in a 5% CO_2_ air atmosphere. Following addition of fresh medium, *Nigella sativa* extract was added at various concentrations namely 0.1%, 0.3%, 0.5, 0.7%, and 1%; and the cells cultured for 24 h and 48 h. *Nigella sativa* extract was not added to the control wells. Changes in cell morphology was imaged using inverted phase contrast optics and the cell viability was assessed by MTT assay.

## Results

Control HepG2 cells maintained their typical morphology and formed a confluent monolayer. In contrast, the cells treated with *Nigella sativa* extract showed varying changes in morphology (cell shrinkage, membrane damage) resulting in cell death and gross decreases in cell numbers starting from 0.3% concentration at both 24 h and 48 h (Figure 1B). MTT assay demonstrated statistically significant decreases in cell proliferation with increasing concentrations of the drug at 24 h and 48 h. The mean decreases in cell proliferation were 18%, 42%, 54%, 56%, and 62% at 24hr; and 23%, 27%, 36%, 38% and 53% at 48hr for the concentrations 0.1%, 0.3%, 0.5%, 0.7% and 1% respectively (Figure 1C, 1D).

## Conclusions

In the present study, the extract of *Nigella sativa* demonstrated inhibition of HepG2 cell line *in vitro*. Our results are in accordance with an earlier study [2] where a different form of *Nigella sativa* extract was found to inhibit the growth and proliferation of the HepG2. We therefore conclude that *Nigella sativa* extract has anticancer properties which needs further exploration and as such we are currently involved in identifying the active ingredient of the extract as well as the underlying molecular mechanism leading to cell death.
